# Supplementary figures and images for: Image-based methods for phenotyping growth dynamics and fitness components in Arabidopsis thaliana
Source: Plant Methods. 2018 Jul 26;14:63. doi: 10.1186/s13007-018-0331-6 (PMC6060534; doi:10.1186/s13007-018-0331-6)

**Figure S1.**

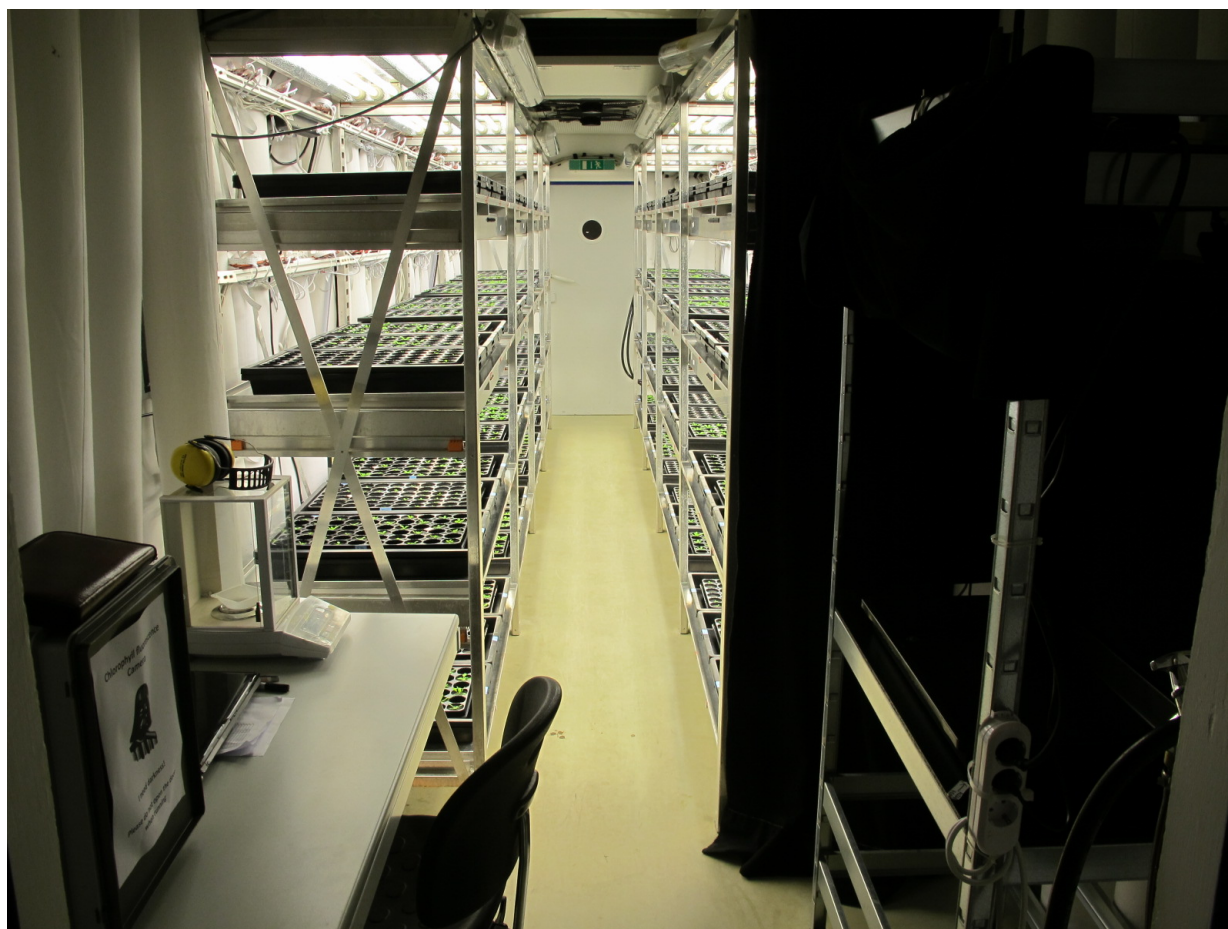

Figure S2.

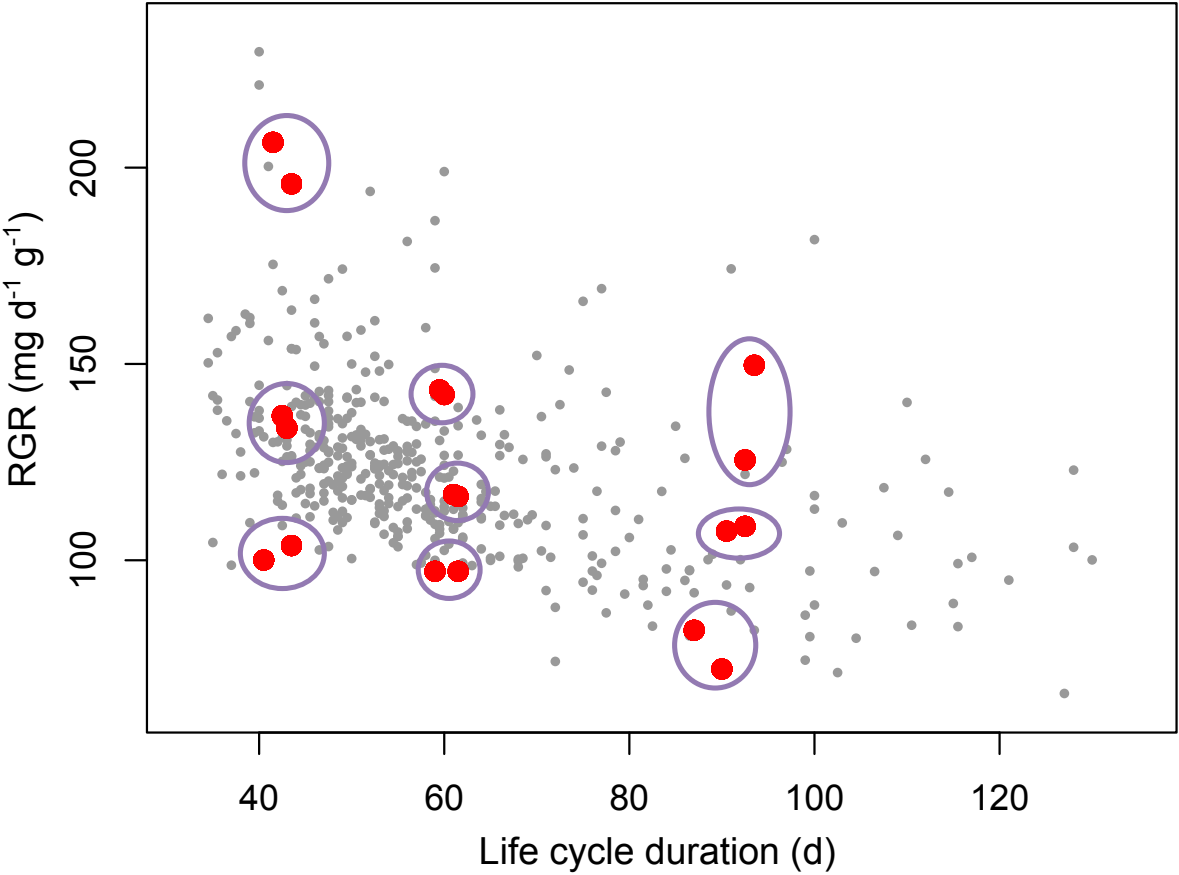

**Figure S3.**

**a**

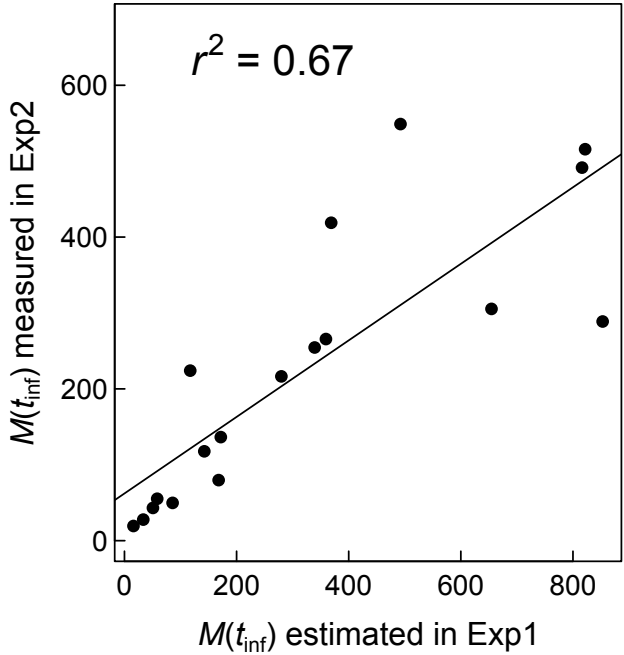

**b**

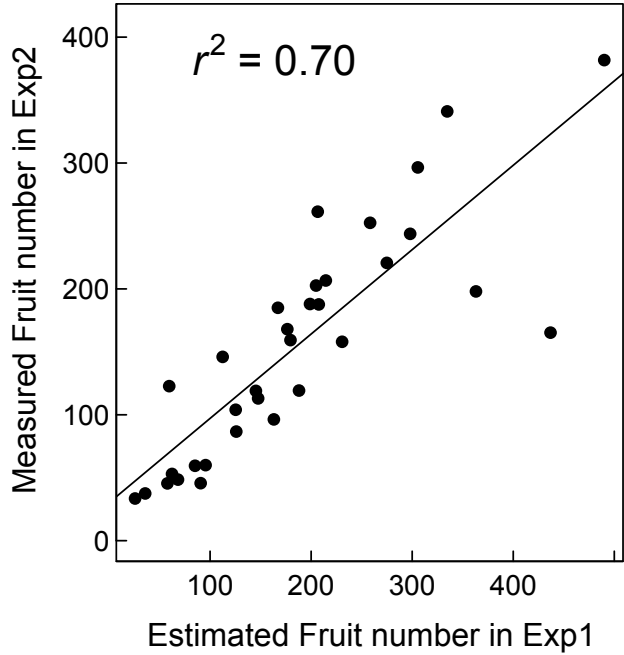

Supplement: Supplementary file 6 — Additional file 6: Figure S1. The RAPA facility. Entrance view of the growth chamber with a zoom on the camera installed between light tubes (top-left panel). On the right is the setup to water the plants and take manual tray picture. Figure S2. Representation of the 18 accessions phenotyped in the second experiment. Nine phenotypic groups represented by the purple circles (three groups of RGR and three groups of growth duration) were selected, each containing two accessions. Figure S3. Inter-experiment reproducibility of rosette dry mass and fruit number estimation. Measured across 18 contrasted accessions. (a) Pearson’s coefficient of correlation (r2) between rosette dry mass M estimated at the inflection point tinf in the first experiment and rosette dry mass M measured at tinf in the second experiment. (b) r2 between the number of fruits estimated in the first experiment and the number of fruits measured in the second experiment. [file 13007_2018_331_MOESM6_ESM.pdf]
